# Perspectives: Digital media use in children with autism: balancing benefits and risks. A nursing perspective

**DOI:** 10.1177/17449871251334419

**Published:** 2025-07-02

**Authors:** Björn Ljungberg, Albert Westergren

**Affiliations:** Licensed Clinical Social Worker, Skåne Region, Habilitation Clinic for Children and Youth, Sweden; Professor, Kristianstad University, Faculty of Health Sciences, Sweden;; Health Care Strategist, Skåne Region, Office for Psychiatry, Habilitation and Technical Aids, Sweden

## Introduction

The rise of digital media has transformed childhood experiences, particularly for children with autism spectrum disorder (ASD), who often gravitate towards screen-based activities. While digital media can offer valuable opportunities for communication, learning and relaxation, there is growing concern about potential negative effects, including problematic gaming, social isolation and sleep disturbances.

Nurses and other professionals working in for instance psychiatry, education and habilitation can encounter families struggling to manage their child’s screen use. Parents report conflicts over digital media use, concerns about excessive gaming, and difficulties setting boundaries. Despite these challenges, families also recognise the positive aspects of digital engagement, particularly in fostering social connections and reducing stress for children with ASD.

This paper explores the benefits and risks of digital media use for children with ASD and outlines practical recommendations for nurses and other professionals supporting families in navigating this complex issue.

This paper is based on a literature review conducted within a development project in a habilitation setting, aimed at identifying evidence-based strategies to best support children and their families in the use of digital media (Ljungberg, 2025).

## The benefits of digital media for children with ASD

### Social communication and connection

Children with ASD often experience difficulties with face-to-face social interactions, making online communication an appealing alternative. Studies show that social media and gaming platforms can provide a structured and predictable environment where children with ASD feel more comfortable engaging with peers where there is less felt need to camouflage their difficulties in offline interaction. Research suggests that children with ASD who use digital media for social purposes report stronger friendships than those who do not (Kuo et al., 2014).

### A sense of control and predictability

Children with ASD frequently exhibit rigid thinking patterns and a preference for predictable environments. Digital media, particularly video games, allow them to engage in structured activities where they can control their surroundings. This can be emotionally regulating, offering a sense of security that may be difficult to achieve in real-world interactions (Pavlopoulou et al., 2022).

### Reducing stress and sensory overload

Digital media can serve as a coping mechanism for children with ASD, helping them manage stress and sensory sensitivities. Parents report that their children use digital media as a form of relaxation after demanding social interactions, particularly in school settings (Mazurek and Wenstrup, 2013; Pavlopoulou et al., 2022).

## The risks of digital media for children with ASD

### Problematic gaming and Internet use

Excessive gaming can become problematic, leading to behaviours resembling Internet Gaming Disorder, recognised in DSM-5 (American Psychiatric Association, 2013). Children with ASD are at an increased risk due to challenges with impulse control and transitioning between activities. Studies show that boys with ASD are more likely to engage in excessive gaming compared to their neurotypical peers (Paulus et al., 2020).

### Sleep disruptions and health consequences

Screen use before bedtime is associated with delayed sleep onset and poorer sleep quality, particularly for children with ASD, who already experience higher rates of sleep disorders (Mazurek et al., 2016). The blue light from screens suppresses melatonin production, and engaging in stimulating activities late at night can make it difficult for children to unwind.

### Social isolation and passive media consumption

While digital media can facilitate communication, children with ASD tend to use it in a more passive manner, such as watching videos rather than actively engaging with others. This can limit opportunities for developing real-world social skills (Jedrzejewska and Dewey, 2022). Additionally, excessive screen time may displace other critical activities such as physical exercise, in-person social interactions and educational tasks.

### Online risks: cyberbullying and exploitation

Children with ASD are more vulnerable to online harassment and exploitation, given difficulties in recognising social cues and distinguishing trustworthy individuals (Nutley and Thorell, 2021). Research suggests that parents of children with ASD report higher levels of difficulty in supervising their child’s online activity, which can increase risks such as cyberbullying, exposure to harmful content and online grooming (Kuo et al., 2014).

### Risk factors for developing an excessive use of digital media

Risk factors include young age, social isolation (Andre et al., 2020), challenges with executive functioning (Turan et al., 2024), difficulty maintaining focus and hyperactivity (Mazurek and Engelhardt, 2013).

## The role of nurses in supporting families

Nurses can play a key role in guiding families towards balanced digital media use. This includes providing evidence-based recommendations, addressing parental concerns and helping families develop individualised strategies based on the child’s specific needs.

### Educating parents on digital media use

Nurses can provide *tailored guidance* on healthy screen habits, helping parents set clear *time limits and boundaries* around digital media. This may include:

- *Establishing screen-free routines*, particularly before bedtime.
- Encouraging the *use of parental controls* and content filters.
- *Promoting active over passive digital engagement* (e.g. creative apps, educational games, social interaction).

### Encouraging balanced activities

To reduce the risk of excessive screen time, nurses can work with families to ensure children engage in a *mix of activities*, including:

- Physical exercise and outdoor play.
- Structured social opportunities (e.g. playgroups, hobby clubs).
- Alternative leisure activities such as music, art or board games.

### Supporting parents in managing conflicts

- *Helping parents set realistic expectations* for screen use.
- Encouraging *open discussions* rather than strict bans.
- Encouraging parents to use *a social strategy* in which they engage with digital media alongside their child, creating an opportunity to validate and acknowledge its importance and thereby foster a positive relationship with it (Kuo et al., 2015).
- Emphasising the *importance of role-modelling balanced media use*.

### Addressing sleep and health concerns

- *No screens at least 30 minutes before bedtime.*
- Using *night mode settings* on devices.
- Establishing a *structured bedtime routine* with relaxing activities.

### Raising awareness about online safety

- Educating parents and children about *cyberbullying and digital risks.*
- Teaching children how to recognise *inappropriate online behaviour*.
- Encouraging *open discussions about online experiences*.

## Conclusion

Digital media plays a complex role in the lives of children with ASD, offering both opportunities and risks. Although it can facilitate social connections, emotional regulation and structured engagement, excessive use may contribute to problematic gaming, sleep disturbances, social isolation and online vulnerability.

Nurses in healthcare, psychiatry, school, community and habilitation settings can have a crucial role in supporting families to establish healthy digital media habits.

## Key takeaways for nursing practice

- Encourage balanced screen use with a mix of digital and offline activities.
- Educate parents on setting limits and fostering positive media habits.
- Support families in reducing bedtime screen exposure for better sleep.
- Raise awareness about online risks and teach safety strategies.
- Adopt a collaborative approach – help parents create a tailored media plan based on the child’s needs.
